# An Unusual Discovery of Multi-Opportunistic Organisms in Gastrointestinal Biopsies of a Patient With Acquired Immunodeficiency Syndrome and Infectious Colitis

**DOI:** 10.7759/cureus.50124

**Published:** 2023-12-07

**Authors:** Chirag Patel, Patricia Le, Malik Salman, Stephen Cavalieri, Joyce Kovar

**Affiliations:** 1 Pathology, Creighton University School of Medicine, Omaha, USA

**Keywords:** opportunistic viral infection, cryptosporidium infection, cytomegalovirus (cmv), opportunistic fungal infection, hiv aids

## Abstract

Patients with acquired immunodeficiency syndrome (AIDS) have an increased risk of infectious colitis. While individual cases of infectious colitis are not rare, co-infections involving multiple opportunistic organisms are uncommon. Here, we present an AIDS patient with concurrent opportunistic gastrointestinal infections resulting in symptomatic infectious colitis.

A 56-year-old woman with AIDS presented to the hospital with diarrhea, abdominal pain, and sepsis. Initial imagining revealed thickening of the colonic wall suggestive of colitis. The initial workup identified the presence of *Campylobacter *and* Cryptosporidium* through the GI Pathogen Multiplex Polymerase Chain Reaction Panel (bioMérieux BioFire®, Salt Lake City, USA), and stool parasite examination also confirmed the presence of *Cryptosporidium.* Despite treatment for these infections, the patient’s diarrhea persisted. The patient had a sigmoidoscopy performed, and the biopsy results revealed the presence of *Histoplasma capsulatum,* cytomegalovirus (CMV)*, *Herpes simplex virus (HSV)*, Cryptosporidium,* and *Campylobacter.* The patient subsequently received appropriate treatment for each infection, leading to the resolution of both colitis and bacteremia.

This case emphasizes the importance of considering multiple pathogens in the management of infectious colitis in patients with AIDS. The presence of one infectious agent does not preclude the presence of additional agents, and a thorough investigation can ensure a definitive diagnosis and optimal treatment for patients.

## Introduction

Patients with acquired immunodeficiency syndrome (AIDS) have a greatly increased risk of various pathogens colonizing the gastrointestinal tract such as *Microsporidia*, *Cryptosporidium*, *Mycobacterium avium complex*, and *Cytomegalovirus *(CMV) [1]. Gastrointestinal symptoms that HIV-positive individuals commonly present are odynophagia, nausea, diarrhea, and abdominal pain [2]. While previous cases have highlighted patients facing opportunistic pathogen infection in their gastrointestinal tract, we present a unique situation involving a patient with concurrent viral, protozoal, and fungal infections not previously reported.

## Case presentation

**Figure 1:**
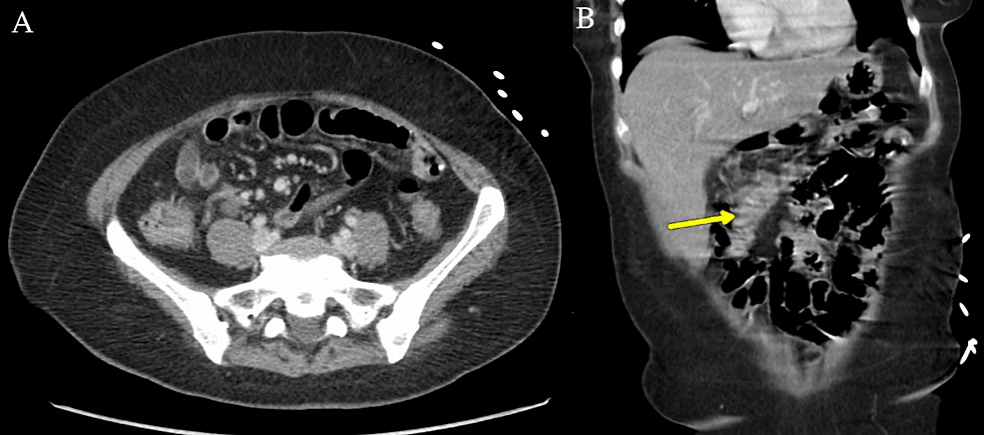
(A) Axial abdominal CT with mild transverse colonic wall thickening suspicious for colitis. (B) Coronal abdominal CT with mild ascending colonic wall thickening.

**Figure 2:**
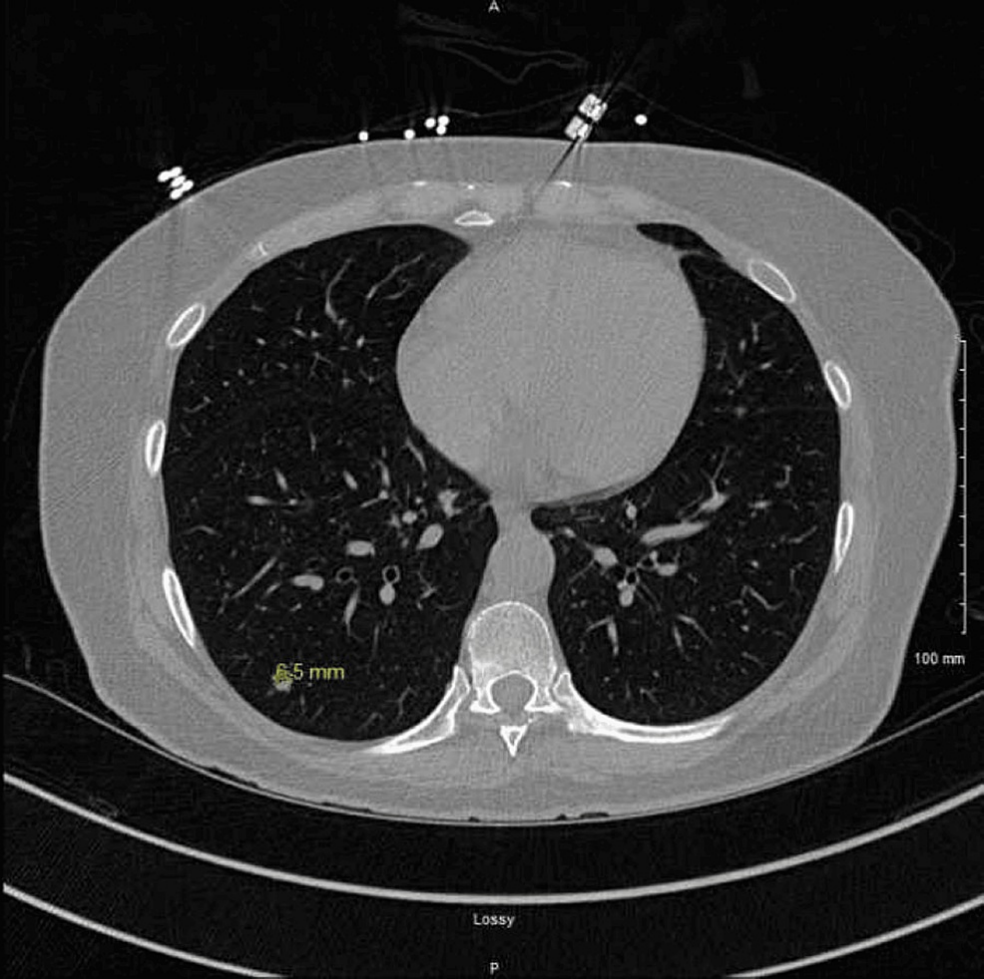
CT chest showing 6.5 mm pulmonary nodule. Multiple nodules were unmarked.

**Figure 3:**
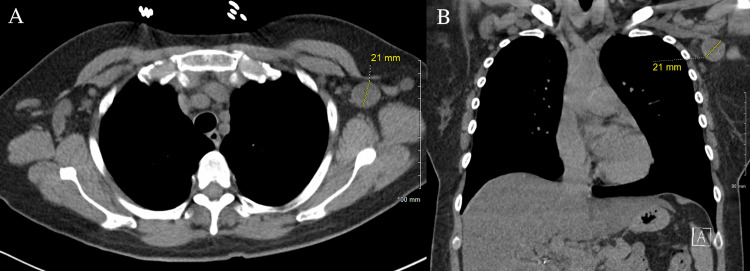
(A) Axial CT chest demonstrating enlarged axillary left lymph node with central low-density concerning for necrosis. (B) Coronal CT chest demonstrating enlarged axillary left lymph node.

We present a 56-year-old woman who was initially transferred from an outside hospital to the academic medical center for higher-level care. She had a complex medical condition including end-stage AIDS with a CD4 count of 6, persistent diarrhea with severe abdominal pain, and sepsis with tachypnea and tachycardia. Abdominal CT imaging displayed ascending and transverse colonic wall thickening suspicious of colitis (Figure 1). Chest CT imaging without contrast showed numerous nodules throughout all lobes of both lungs with the greatest measuring 6.5 mm, as well as markedly enlarged bilateral axillary lymph nodes (Figures 2-3).

Based on chest CT imaging findings, an etiologic diagnosis of the pulmonary nodules was sought. Tuberculosis was initially suspected because she had visited El Salvador one month prior, though acid-fast stains were negative. The nodules were found to be secondary to *Histoplasma capsulatum* confirmed by cultures of bronchoscopy samples. She was also found to have disseminated histoplasmosis when an axillary lymph node biopsy showed findings most consistent with Histoplasma lymphadenitis. In addition, urine histoplasma antigen was positive. Additional diagnostic tools were used to evaluate the causative pathogens of her sepsis and diarrhea. GI Pathogen Multiplex PCR Panel (bioMérieux BioFire®, Salt Lake City, USA) identified the presence of *Campylobacter *and *Cryptosporidium*, and stool parasite exam also confirmed the presence of *Cryptosporidium*. Due to the patient’s nonfunctional gastrointestinal tract, the patient required a peripherally inserted central catheter (PICC) line and total parenteral nutrition (TPN) for two and a half weeks.

**Figure 4:**
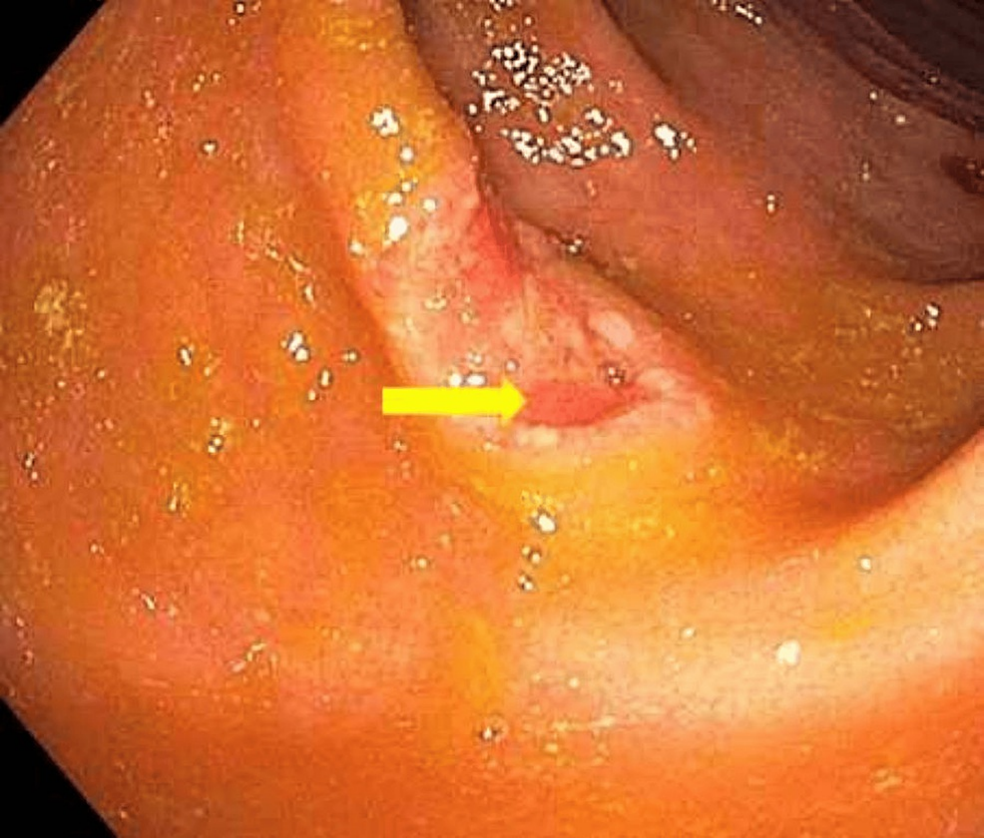
A 75mm solitary non-bleeding ulcer in the distal transverse colon with heaped-up margins.

**Figure 5:**
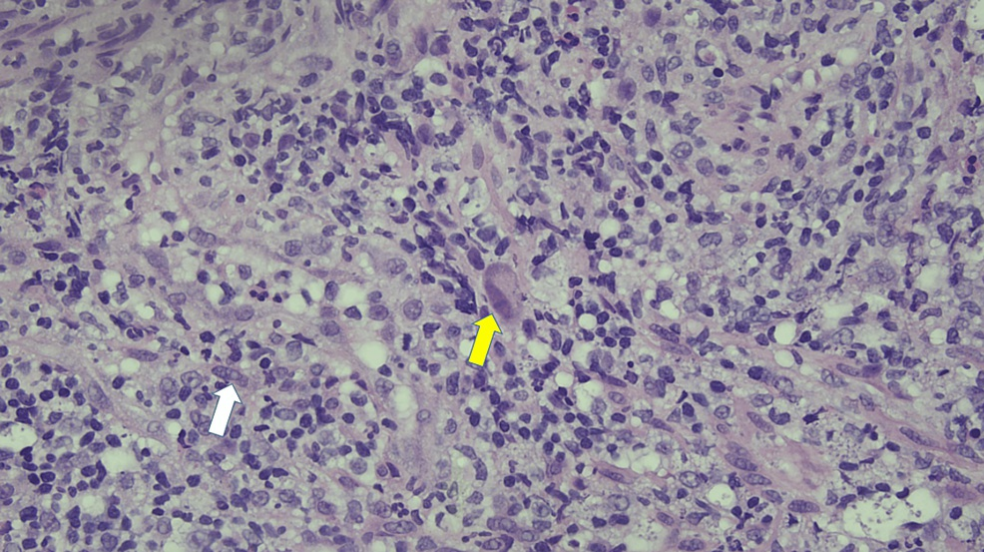
Cytomegalovirus- and herpes simplex virus-infected cells shown in yellow arrow and white arrow, respectively, on ulcer of left descending colon on hematoxylin and eosin stain at 40x magnification.

**Figure 6:**
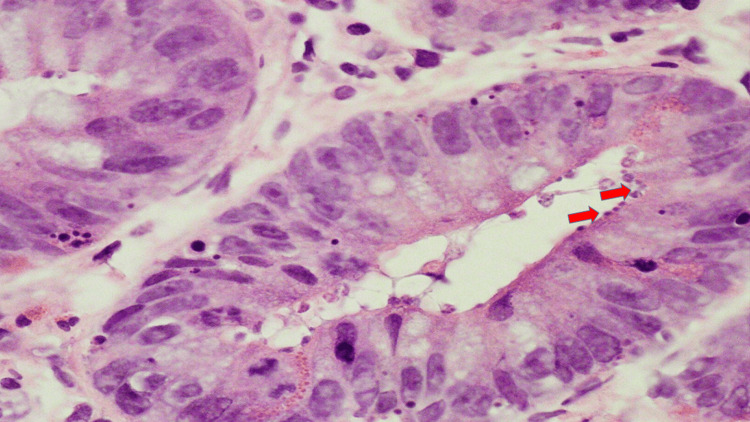
Cryptosporidium organisms in gastrointestinal biopsy of the right colon on hematoxylin and eosin stain at 100x magnification with red arrows highlighting representative organisms.

**Figure 7:**
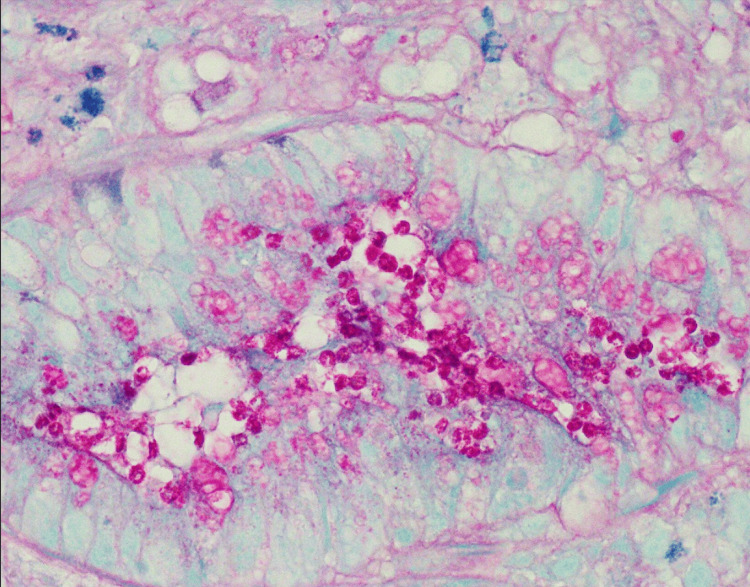
Cryptosporidium organisms in gastrointestinal biopsy of the right colon on periodic acid-Shiff stain at 100x magnification.

**Figure 8:**
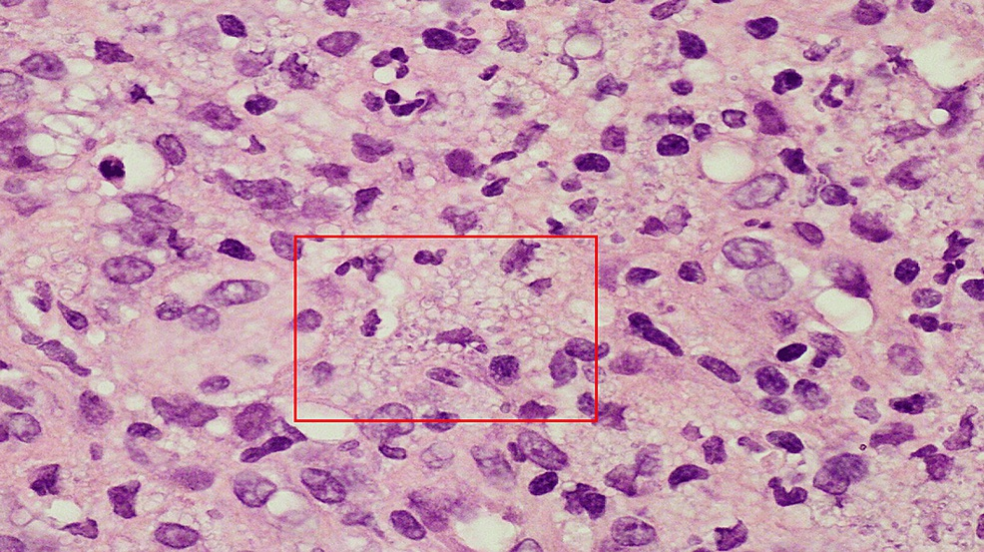
Histoplasma capsulatum organisms in gastrointestinal biopsy of the splenic flexure of the colon on hematoxylin and eosin stain at 100x magnification with the red box highlighting representative cluster.

**Figure 9:**
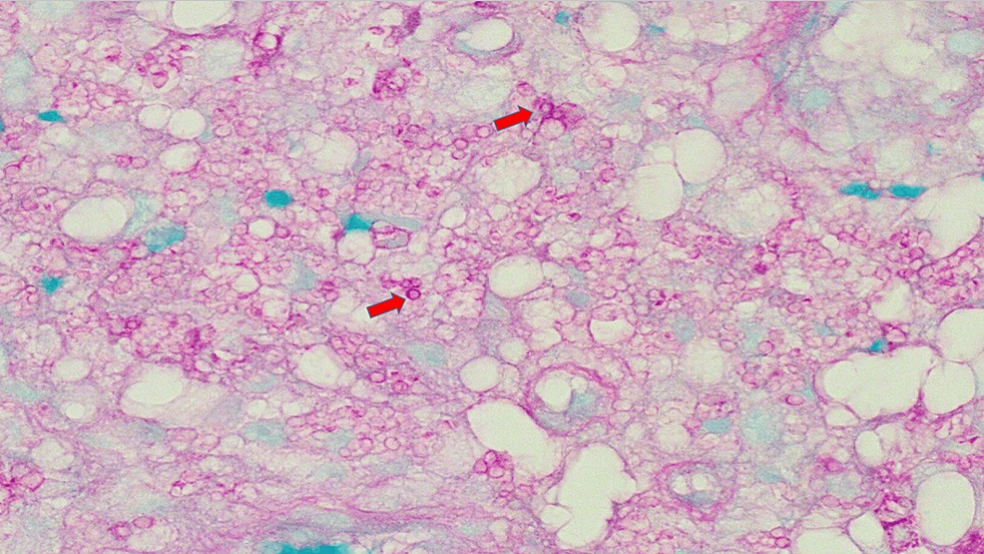
Histoplasma capsulatum organisms in gastrointestinal biopsy of the splenic flexure of the colon on periodic acid-Shiff stain at 100x magnification with red arrows highlighting representative organisms.

**Figure 10:**
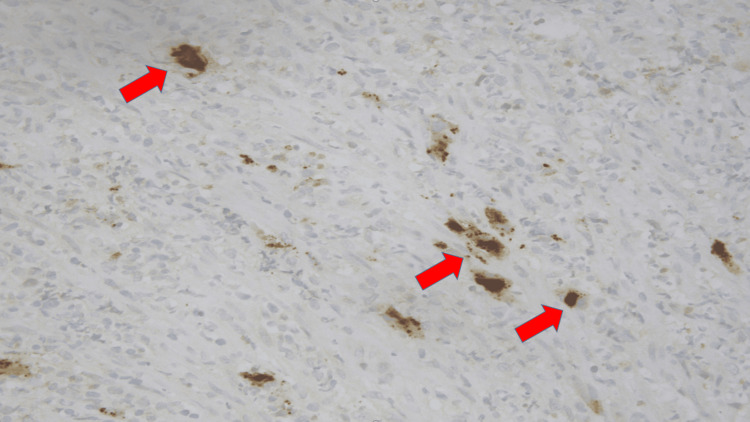
Immunohistochemical stain for herpes simplex virus (HSV) on ulcer of left descending colon at 40x magnification with red arrows highlighting HSV.

**Figure 11:**
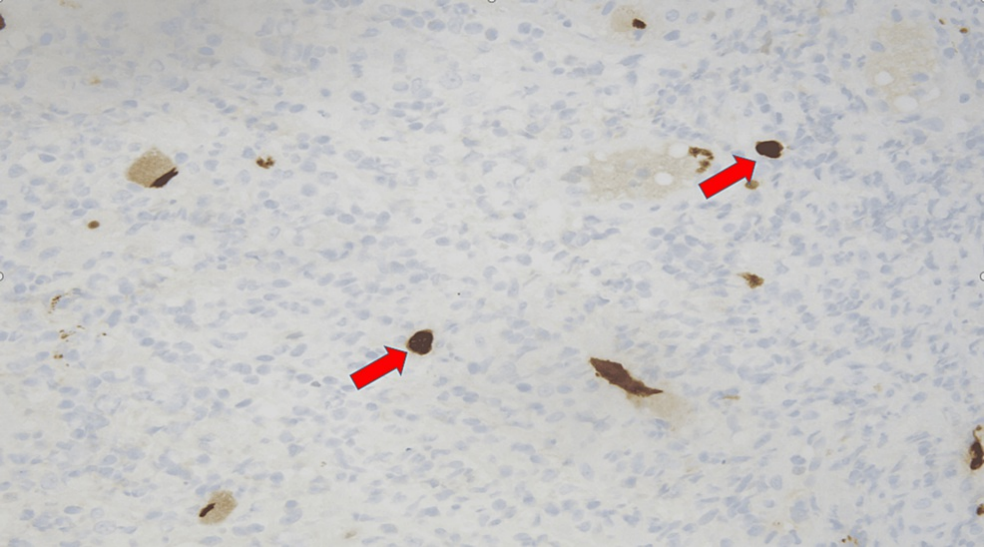
Immunohistochemical stain for cytomegalovirus (CMV) on ulcer of left descending colon at 40x magnification with red arrows highlighting CMV.

Nitazoxanide was administered for her *Cryptosporidium *colitis and itraconazole for her disseminated histoplasmosis. Due to persistent diarrhea, a flexible sigmoidoscopy was performed which was remarkable for a 7.5-centimeter ulcer in the distal transverse colon and a one-centimeter ulcer in the mid descending colon (Figure 4). Through various staining methods, additional microorganisms were identified that were not detected on prior laboratory workups. Hematoxylin and eosin as well as Periodic Acid Schiff stains revealed the presence of *H. capsulatum* and *Cryptosporidium *(Figures 5-9). Immunostains on biopsies at ulcer sites were strongly positive for both Herpes simplex virus (HSV) and CMV (Figures 10-11).

Her hospital stay was complicated by *Staphylococcus epidermidis* bacteremia likely from her PICC line and she was started on vancomycin. Due to improving gastrointestinal function, interventional radiology placed a percutaneous endoscopic gastrostomy tube. After two months of hospital stay, the patient was discharged with full resolution of colitis and bacteremia.

## Discussion

When presenting with persistent diarrhea, the consideration of a wide variety of opportunistic infections should be considered for immunocompromised patients. Although the risk of opportunistic infections is dramatically increased in these patients, concurrent multiple coinfections remain rare. For example, disseminated histoplasmosis occurs in individuals in endemic areas in 5-25% of patients with advanced AIDS [3]. Meanwhile, *Cryptosporidium *colitis occurs in 5-10% of AIDS patients and CMV colitis occurs in approximately 7% of AIDS patients [4,5]. Few examples of HSV colitis have been described in the literature [6]. One case has been described demonstrating a coinfection of HSV, CMV, and histoplasmosis located in the oral cavity [7]. Furthermore, our review of the literature found no coinfections of *Cryptosporidium*, *H. capsulatum*, CMV, and HSV-causing colitis.

Due to nonspecific clinical manifestations such as fever, diarrhea, weight loss, and abdominal pain, the etiologies of infectious colitis are not initially obvious. Microorganisms such as *Cryptosporidium*, *H. capsulatum*, CMV, and HSV are potential causes of these symptoms. Colonoscopy findings are also non-specific and can show signs of ulceration, thickened walls, and plaques [8]. CT imaging may suggest infectious colitis with patients demonstrating colonic wall thickening and edema. Pericolonic fat inflammation and ascites may also be present. Additionally, infectious organisms affect different segments of the bowel. For example, CMV typically presents in a diffuse pattern, while HSV typically affects the rectosigmoid portion, and *H. capsulatum* typically affects the ileum [9,10]. While imaging may narrow the differential for the cause of infectious colitis, visualization of diagnostic gastrointestinal biopsies with various stains can provide the most definitive answer.

Patients typically present with one etiology causing infectious colitis. However, multiple different organisms causing colitis in a patient can complicate management. A biopsy is rarely needed, as other measures such as stool cultures, imaging, and empiric treatment can lead to resolution before more invasive procedures are necessary. In the case of our patient, the non-invasive procedures did not lead to a complete and definitive diagnosis of her colitis. Her symptoms persisted after receiving incomplete therapy for her colitis. These circumstances required further investigations including a biopsy to determine a definitive diagnosis. Biopsy and immunohistochemical staining can provide a definitive diagnosis and aid in the treatment of patients with worsening colitis.

## Conclusions

This patient’s hospital course reveals the importance of correlating an AIDS diagnosis with the evaluation of potential pathogens present in gastrointestinal biopsies. The patient’s persistent diarrhea is likely due to the incomplete initial treatment regimen prior to colonoscopy. Through stool parasite exam and GI Pathogen Multiplex PCR Panel, only *Campylobacter *and *Cryptosporidium *were identified. After identifying the presence of CMV and HSV from biopsy immunostains, clinicians added ganciclovir to her treatment regimen and her diarrhea subsequently resolved.
